# Modulation of Aromatic Amino Acid Metabolism by Indigenous Non-*Saccharomyces* Yeasts in Croatian Maraština Wines

**DOI:** 10.3390/foods13182939

**Published:** 2024-09-17

**Authors:** Ana Boban, Urska Vrhovsek, Andrea Anesi, Vesna Milanović, Jasenka Gajdoš Kljusurić, Zvonimir Jurun, Irena Budić-Leto

**Affiliations:** 1 Institute for Adriatic Crops and Karst Reclamation, 21000 Split, Croatia; aboban@krs.hr (A.B.); zjurun@krs.hr (Z.J.); 2 Metabolomics Unit, Research and Innovation Centre, Fondazione Edmund Mach, 38010 San Michele all’Adige, Italy; urska.vrhovsek@fmach.it (U.V.); andrea.anesi@fmach.it (A.A.); 3 Department of Agricultural, Food and Environmental Sciences, Polytechnic University of Marche, 60131 Ancona, Italy; v.milanovic@univpm.it; 4 Faculty of Food Technology and Biotechnology, University of Zagreb, Pierottijeva 6, 10000 Zagreb, Croatia; jgajdos@pbf.hr

**Keywords:** yeast isolates, tryptophan, sterile grape juice, UHPLC-MS/MS, monoculture, sequential fermentation

## Abstract

This study aimed to provide novel information on the impact of indigenous non-*Saccharomyces yeasts*, including *Metschnikowia chrysoperlae*, *Metschnikowia sinensis*/*shanxiensis*, *Metschnikowia pulcherrima*, *Lachancea thermotolerans*, *Hanseniaspora uvarum*, *Hanseniaspora guilliermondii*, and *Pichia kluyveri*, on metabolites related to the metabolism of tryptophan, phenylalanine, and tyrosine. The experiment included two fermentation practices: monoculture and sequential fermentation with commercial *Saccharomyces cerevisiae*, using sterile Maraština grape juice. A targeted approach through ultrahigh-resolution liquid chromatography associated with mass spectrometry was used to quantify 38 metabolites. All the indigenous yeasts demonstrated better consumption of tryptophan in monoculture than in interaction with *S. cerevisiae*. *M. sinensis*/*shanxiensis* was the only producer of indole-3-carboxylic acid, while its ethyl ester was detected in monoculture fermentation with *H. guilliermondii*. *H. guilliermondii* consumed the most phenylalanine among the other isolates. 5-hydroxy-L-tryptophan was detected in fermentations with *M. pulcherrima* and *M. sinensis*/*shanxiensis*. *M. pulcherrima* significantly increased tryptophol content and utilised tyrosine in monoculture fermentations. Sequential fermentation with *M. sinensis*/*shanxiensis* and *S. cerevisiae* produced higher amounts of N-acetyl derivatives of tryptophan and phenylalanine, while *H. guilliermondii*-*S. cerevisiae* fermentation resulted in wines with the highest concentrations of L-kynurenine and 3-hydroxyanthranilic acid. *P. kluyveri* produced the highest concentration of N-acetyl-L-tyrosine in monoculture fermentations. These findings highlight the different yeast metabolic pathways.

## 1. Introduction

Wine production involves biochemical processes where yeasts metabolise grape sugars into ethanol, carbon dioxide, and other volatile and non-volatile compounds, which contribute to wine aroma [1]. To achieve this, yeasts utilise the nutrients present in grape juice to evaluate the growth and produce different metabolites [2]. The main sources of yeast-assimilable nitrogen in grape must are ammonium and amino acids. As a result, many aroma compounds are directly related to nitrogen metabolism, especially to aromatic amino acids.

Aromatic amino acids are catabolised by the transamination of the amino group, forming alpha-keto acids, which are then decarboxylated to the aldehydes [3]. The aromatic amino acids, including tryptophan (TRP), phenylalanine (PHE), and tyrosine (TYR), can be consumed by yeasts but are less preferable [4]. Sprenger et al. [5] first reported the capability of *Saccharomyces cerevisiae* to metabolise L-tryptophan. Rodríguez Naranjo et al. [6] determined the capacity of various *Saccharomyces* strains to form melatonin during growth and alcoholic fermentation. In addition to *S. cerevisiae*, there is growing interest in applying non-*Saccharomyces* yeasts in the winemaking procedure, mainly due to their enzymatic activity [7,8], which enhances wine aromas [9]. These species can positively impact winemaking by producing high amounts of aromatic compounds, such as aromatic alcohols, ethyl esters, and acetate esters, and realising terpenic compounds [10,11,12]. Unlike *S. cerevisiae*, little is known about the behaviour of non-*Saccharomyces* yeasts during wine fermentation, and their utilisation/production of amino acids and their derivates has been poorly explored. The catabolites of TRP, TYR, and PHE include several sensorial and biologically active compounds, such as tryptophol (TOL), tyrosol (TYL), hydroxytyrosol (OH-TYL), kynurenic acid (KYNA), kynurenine (KYN), indole acetic acid (IAA), indole lactic acid (ILA), and ethyl esters of TRP (TRP-EE) and TYR (TYR-EE) [13]. Through the Ehrlich pathway, TRP, TYR, and PHE produce higher aromatic alcohols, like TOL, TYL, and phenylethanol, whose biosynthesis positively correlates with ethanol stress-tolerant yeast. Optimal concentrations of higher alcohols positively impact wines with floral character [14,15]. Additionally, TRP metabolism by yeast can contribute to wine aroma directly by biotransforming odourless metabolites into flavour-active compounds, such as methyl mercaptan and indole, and indirectly through chemical reactions in wine, producing substances like 2-aminoacetophenone (2AA), which is known as an untypical ageing compound [16].

Several studies have investigated the formation of L-TRP derivatives by the yeast strains because of their ability to rapidly complete fermentation and their wide application in white, rosé, and red winemaking. Fernández-Cruz et al. [17] investigated the synthetic pathway of melatonin and its intermediates in commercial *Torulaspora delbrueckii*, *M. pulcherrima*, and *S. cerevisiae* strains. Fernández-Cruz et al. [18] reported the impact of different grape varieties, and the commercial yeast strains *T. delbrueckii* and *S. cerevisiae*, on the formation of L-TRP metabolites. Furthermore, the synthesis pattern of tryptophol, tyrosol, and phenylethanol, depending on glucose, nitrogen, and aromatic amino acid availability, has been evaluated for the first time in indigenous *T. delbrueckii*, *M. pulcherrima*, *H. uvarum*, and *Starmellera bacillaris* strains [19]. The most comprehensive study was conducted by Álvarez-Fernández et al. [13] using ultrahigh-resolution liquid chromatography associated with the mass spectrometry method (UHPLC-MS/MS) to monitor the metabolites related to aromatic amino acid intra- and extracellular metabolism during the alcoholic fermentation in the synthetic must of two *S. cerevisiae* yeast strains and one *T. delbrueckii* yeast strain. Some of these results were confirmed in real grape juice fermentations of Chardonnay and Pinot Gris, where Álvarez-Fernández et al. [15] also monitored the metabolites of the aromatic amino acid from both intra- and extracellular metabolisms. Yilmaz and Gökmen [20] reported the effect of several commercial non-*Saccharomyces* yeasts on the formation of amino acid derivates in red and white wines.

All the mentioned studies focus on commercial and similar yeast strains, mainly *M. pulcherrima* and *T. delbrueckii.* The goal of this work was to provide the first insights into the impact of indigenous non-*Saccharomyces* yeasts isolated from the Croatian white grape variety Maraština on the metabolism of aromatic amino acids, such as TRP, PHE, and TYR, during the fermentation of filter-sterilised Maraština grape must. In detail, a targeted UHPLC-MS/MS approach was used to test the impact of *H. uvarum*, *H. guilliermondii*, *L. thermotolerans*, *M. pulcherrima*, and *P. kluyveri*, as well as two species not previously studied in oenological environments—*M. chrysoperlae* and *M. sinensis/shanxiensis*—on wine aromatic amino acid metabolism in both monoculture and sequential fermentations with *S. cerevisiae*. The results were also compared with fermentations performed using commercially available strains of *M. pulcherrima*, *L. thermotolerans*, and *S. cerevisiae*. This approach allowed an accurate assessment of each yeast’s performance in different fermentation practices. These findings will enhance our understanding and monitoring of how indigenous non-*Saccharomyces* yeasts contribute to wine characteristics through TRP, PHE, and TYR metabolisms.

## 2. Materials and Methods

### 2.1. Chemicals

All the chemicals listed in Table S1 were purchased from Sigma-Aldrich (Milan, Italy), except for 3-methoxy-*p*-tyramine, 5-hydroxy-L-tryptophan, L-tryptophan-d5, kynurenic acid, and xanthurenic acid, which were purchased from Spectra-2000 (Rome, Italy), and 5-hydroxytryptophol, which was purchased from ChemSpace (Riga, Latvia). LC-MS grade acetonitrile and the formic acids were purchased from Sigma-Aldrich (Milan, Italy). Ultrapure Milli-Q deionised water was obtained in-house from Elix (Merck-Millipore, Milan, Italy).

### 2.2. Indigenous Non-Saccharomyces Yeast

The alcoholic fermentations of white Maraština must were performed with seven indigenous non-*Saccharomyces* yeasts from an established yeast collection of the Institute for Adriatic Crops and Karst Reclamation (Split, Croatia). The yeasts, including *M. chrysoperlae* K-11 (*Mc*), *M. sinensis/shanxiensis* P-7 (*Ms*), *M. pulcherrima* K-6 (*Mp*), *L. thermotolerans* P-25 (*Lt*), *H. uvarum* Z-7 (*Hu*), *H. guilliermondii* N-29 (*Hg*), and *P. kluyveri* Z-3 (*Pk*), were selected according to their enzymatic and oenological properties, as previously reported by Milanović et al. [8]. The yeasts, preserved in glycerol stocks at −80 °C, were inoculated into YPD broth (10 g/L yeast extract, 20 g/L peptone, 20 g/L dextrose). They underwent two consecutive rounds of preculturing at 25 °C with continuous agitation at 2000 rpm for 24 h in an orbital incubator (Stuart SI500—Incubator, TecQuipment Ltd., Nottingham, UK). The yeast extract, peptone, and bacteriological agar used to prepare the yeast peptone dextrose (YPD) agar/broth as growth media for the yeasts were purchased from Biolife Italiana S.r.l (Milan, Italy), along with bacteriological dextrose supplied by Oxoid (Hampshire, UK). The biomass was collected by centrifugation (Hettich^®^ Universal 320/320R centrifuge Andreas Hettich GmbH & Co., Tuttlingen, Germany) at 1520× *g* at 4 °C for 5 min. The supernatant was carefully removed, and the cell pellet was resuspended in a sterile physiological solution (0.85% NaCl, *w*/*v*). The yeast cell concentration was spectrophotometrically measured at 600 nm using a Varian Cary^®^ 50 UV-Vis Spectrophotometer (Agilent Technologies Inc., Santa Clara, CA, USA). The commercial yeasts were rehydrated according to the manufacturer’s protocols under sterile conditions and prepared for inoculation as described for the indigenous yeasts.

### 2.3. Primary Grape Processing

The experiment was performed on the Croatian white grape variety Maraština (*Vitis Vinifera* L.). The Maraština grapes were sourced from a vineyard in Plastovo (Skradin, 43°52′49″ N 15°55′29″ E), part of the North Dalmatia wine subregion. Harvesting took place on 17 September 2022, at technological maturity (glucose 118.53 g/L, fructose 116.30 g/L). Following harvesting, the grapes underwent primary processing, including treatment with potassium metabisulfite, to achieve a total SO_2_ concentration of approximately 50 mg/L. Subsequently, the grape must was cold-stabilised for 24 h at 4 °C and processed further through sterile filtration using a PALL filler (0.45 µm). The pH value of the grape juice was 3.35, and the total acidity was measured at 4.33 g/L, with malic acid contributing 1.38 g/L. These measurements were obtained using the Lyza 5000 Wine analyser (Anton Paar GmbH, Graz, Austria).

### 2.4. Fermentation Procedure and Sampling

Laboratory-scale fermentations were conducted in Erlenmayer flasks closed with porous cellulose caps, containing 500 mL of sterile Maraština grape juice, at 20 °C in three replications. To optimise nutrients for yeasts in fermentation, the yeast assimilable nitrogen concentration of the grape juice was adjusted to 250 mg/L by adding di-ammonium hydrogen phosphate (VWR International, Radnor, PA, USA). The experimental design included seven non-*Saccharomyces* yeasts in monoculture fermentation and their sequential fermentation with *Sc*. The non-*Saccharomyces* yeast strains were inoculated at a concentration of approximately 5 × 10^6^ cells/mL into sterile grape juice. In sequential fermentations, *Sc* was inoculated at the same concentration when the ethanol concentration reached between 2 and 3% *v*/*v*. Analogously, the commercial yeasts purchased from Lallemand Inc. (Montreal, QC, Canada), *S. cerevisiae* EC 1118 (*Sc*), *L. thermotolerans* Octave (*Lt* Octave), and *M. pulcherrima* Flavia (*Mp* Flavia), were used as the controls for both the monoculture and the sequential fermentations.

The sampling points for ultrahigh liquid chromatography (UHPLC) analysis were at the beginning of fermentation (grape juice) and at the end of the fermentation trials, as defined by constant reducing sugar concentrations, which were typically below 5 g/L. Then, 15 mL quantities of young wine samples were transferred into tubes under sterile conditions, centrifuged at 1520× *g* for 5 min at 4 °C to separate cells from extracellular contents (Hettich^®^ Universal 320/320R centrifuge, Andreas Hettich GmbH & Co., Tuttlingen, Germany), and stored at −80 °C until UHPLC analysis.

### 2.5. UHPLC-MS/MS Analysis

Before UHPLC-MS/MS analysis, the samples were diluted ten times with deionised water and spiked with L-tryptophan-d5 as an internal standard. Subsequently, the samples were filtrated using 13 mm VWR syringe filters with 0.45 μm polytetrafluorethylene (PTFE) membranes directly into an autosampler vial.

The targeted method was adopted from that of Arapitsas et al. [21], with minor modifications. UHPLC-MS/MS was conducted on an AB Sciex 6500+ triple quadrupole-linear ion trap (QqQ) coupled to a Shimadzu LC-30 AD pump (AB Sciex, Milan, Italy) to separate 37 metabolites. Chromatographic separation was performed on the Acquity Premier HSS T3 (2.1 × 50 mm, 1.8 μm particle size) (Waters, Milan, Italy). The mobile phases were 0.1% formic acid in water (A) and 0.1% formic acid in acetonitrile (B) with a flow rate of 0.5 mL/min. The gradient was programmed as follows: 1% B (0 min); 5% B (1 min); 12.5% B (1.5 min); 30% B (2 min); 45% B (3 min); 55% B (3.5 min); 75% B (4 min); 95% B (4.5–5 min); and 1% B (5.1 min). The column temperature was kept at 40 °C. The injection volume was 10 μL to allow the quantitation as a function of the concentration of metabolites. Table S1 provides the multiple reaction monitoring (MRM) parameters and retention time for each metabolite. Further details can be found in Anesi et al. [22].

### 2.6. Statistical Analysis

The software Statistica (StatSoft, Tulsa, OK, USA) v.12.0 was used for the statistical analyses. The data underwent the Kolmogorov–Smirnov test to check the normal distribution. Differences in the metabolites related to the aromatic amino acid metabolism of TRP, PHE, and TYR between the yeast strains and fermentation practices were tested by two-way ANOVA analysis, with differences considered significant when *p* < 0.05. Hierarchical cluster analysis, generated by the Ward algorithm and Euclidean distance, was performed using MetaboAnalyst 5.0 [23] (accessed on 6 July 2024) to display differences among the amino acids and their derivates due to the yeast strains for the two fermentation practices. Debiased sparse partial correlation (DSPC) networks were constructed using the Network Analysis function in MetaboAnalyst 5.0, based on the method described by Basu et al. [24].

## 3. Results and Discussion

Among the 38 metabolites which were analysed by the targeted UHPLC method, 22 compounds were detected and quantified in the grape juice, and 26 were found in the final wines produced by two different inoculation practices: monoculture and sequential fermentations of seven non-*Saccharomyces* isolates (Table 1). The metabolites related to TRP, PHE, and TYR metabolism in Table 1 are sorted in ascending order based on the retention time of the measured MS/MS spectrum (Table S1). The results were compared between the yeast isolates and control treatments in the monoculture and sequential fermentations (rows), as well as the different fermentation practices for each yeast isolate (columns).

### 3.1. Extracellular Metabolic Profile of Yeast Non-Saccharomyces Isolates in Maraština Wines Produced by Different Inoculation Practices

**Figure 1:**
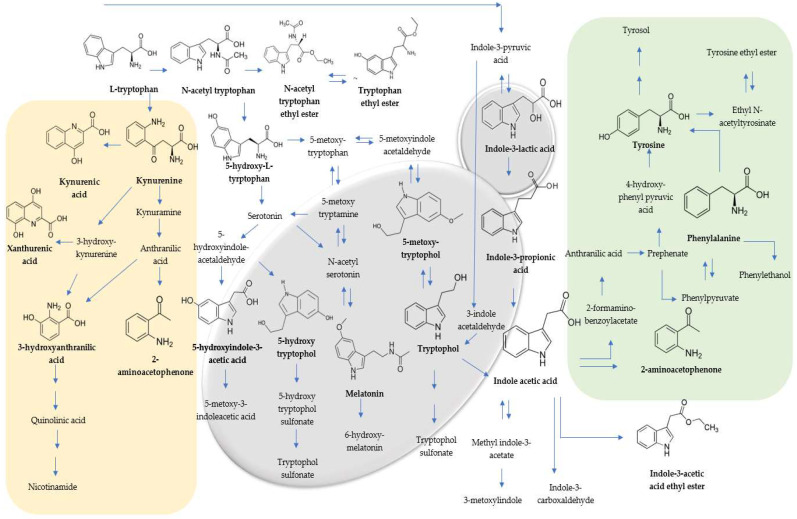
Scheme of the proposed pathway for tryptophan (TRP), tyrosine (TYR), phenylalanine (PHE), and related compounds. The left box outlines the kynurenine pathway (yellow), while the right box highlights the reactions of compounds related to TYR and PHE metabolism (green). The bold names with chemical structures indicate the compounds identified in this study. The scheme is taken from Álvarez-Fernández et al. [13], with modifications.

TRP metabolism involves the decomposition of the indole ring and the formation of kynurenine and its derivatives via a kynurenic pathway. Other compounds that retain the indole ring produce chemical messengers of the indolamine family, including melatonin (MEL) (Figure 1) [25]. Approximately 95% of tryptophan catabolism proceeds via the kynurenine pathways in humans [26], but these pathways have also been reported in yeasts [27].

The initial TRP concentration in grape juice depends on the type of grape cultivar [28,29]. The Maraština grape juice used as the substrate for the fermentations was characterised by a TRP concentration of 2124.93 µg/L. At the end of all the monoculture fermentations, performed with indigenous non-*Saccharomyces* yeasts and commercially available control strains, including *Sc*, the TRP concentration was below 0.08 µg/L; no significant differences among them were shown. On the other hand, all the sequential fermentations resulted in TRP concentrations ranging from 41.72 µg/L (*Pk-Sc*) to 66.64 µg/L (*Hg-Sc*). This indicates that the non-*Saccharomyces* yeasts, in combination with the *Sc* yeasts, did not fully metabolise this amino acid. The significant impact of the sequential fermentations for four yeasts, including *Mp-Sc*, *Lt-Sc*, *Hu-Sc*, and *Hg-Sc*, statistically differed from the *Sc* control and their respective monoculture fermentations, *Mp*, *Lt*, *Hu*, and *Hg*. According to Fernandez-Cruz et al. [18], the final TRP concentrations in two white wines produced by commercial *S. cerevisiae* ranged from 4.20 µg/L for Vijiriega to 1902 µg/L for Moscatel. Tryptophanase can convert the odourless substrate L-tryptophan (TRP) into the odorous products methyl mercaptan and indole [3].

The kynurenine pathway converts TRP into neuroactive metabolites, such as L-Kynurenine (KYN) [30]. KYN can further metabolise into kynurenic acid (KYNA), 3-hydroxyanthranilic acid (OH-ANT), and 3-hydroxykynurenine (3OH-KYN). In the monoculture fermentations, *Mc*, *Ms*, and *Hu* increased the concentration of KYN (4.69–5.34 µg/L) from the initial fermentation stage (3.30 µg/L). Among the tested yeasts, the control *Lt* Octave produced the highest quantity of KYN (10.62 µg/L), followed by indigenous *Ms* (5.34 µg/L). Additionally, there was a notable difference between the indigenous *Lt* and commercial *Lt* Octave strains, where the control strain resulted in a concentration three times higher than that in the grape juice. The significant impact of the fermentation practices was observed in the increased concentrations of KYNA in the white Maraština wines. Sequential fermentation involving *Mp* and *Hg* with *Sc* resulted in statistically higher concentrations of KYNA (299.93–303.24 µg/L) compared to their pure fermentations (73.95–122.82 µg/L). The lower TRP content in wines obtained through pure fermentations might contribute to the lack of kynurenic acid formation, as previously reported by Yilmaz et al. [20]. A previous study by Turska et al. [31] reported a much lower content of KYNA in white wines, ranging from 14 to 17 µg/L. Subsequently, 3-hydroxyanthranilic acid (OH-ANT) was not detected in Maraština grape juice. In the final wines, the highest concentrations were observed with *Hg* isolates during its sequential fermentation with *Sc*, reaching 89.76 µg/L. This was statistically higher compared to the *Sc* control fermentation (34.55 µg/L) and the *Hg* monoculture fermentations (34.52 µg/L). OH-ANT can further convert into other compounds, such as quinolinic acid, and has antioxidant properties that positively impact health [32]. The Maraština grape juice contained 6.90 µg/L of 3-hydroxykynurenine (3OH-KYN), but its concentration slightly decreased in the final wines. Interestingly, 3OH-KYN was not detected in the pure fermentations of *Hu* and the sequential fermentation of *Pk-Sc*, possibly due to further conversion or concentrations falling below the detection limit [22]. The concentrations did not significantly differ among the investigated non-*Saccharomyces* yeast strains compared to the controls and the different inoculation practices. The absence of 3OH-KYN in the *Hu* monoculture fermentation and *Pk-Sc* fermentations aligns with the findings of Yilmaz and Gökmen [20], who did not identify 3OH-KYN in white Çavuş wines and red Cardinal wines. Its antioxidant properties may contribute to the stability and quality of the wine. Xanthurenic acid (XA) is a product of the tryptophan–kynurenine pathway. Among the monoculture fermentations, *Mp* yielded the highest concentration of XA (17.12 µg/L), followed by *Pk* (13.72 µg/L), which was statistically significant compared to the control fermentations with *Mp* Flavia (6.41 µg/L) and *Sc* (5.23 µg/L). The impact of the inoculation treatments was observed with the *Ms* and *Hg* yeast isolates, where the sequential fermentations (17.48 and 13.68 µg/L) of these two yeasts resulted in a significantly higher amount of XA compared to the monoculture fermentations (9.43 µg/L and 6.51 µg/L) and the *Sc* control. All the non-*Saccharomyces* yeast isolates metabolised XA more effectively than *Sc*, regardless of the fermentation treatment. The initial concentration of XA (13.41 µg/L) changed differently across the yeast treatments. While the concentration mostly decreased, the sequential fermentations with *Ms-Sc* and *Hg-Sc* and the monoculture fermentations with *Mp* and *Pk* showed an increase in XA levels. 2-aminoacetophenone (2AA) can be produced from anthranilic acid in the kynurenic pathway as well as by the degradation of indole-acetic acid (IAA) [16]. This study found no significant impact from the type of yeast or inoculation strategy used. In the context of wine, 2AA is directly associated with an untypical ageing off-flavour. The *Sc* fermentations increased the initial concentration (0.96 µg/L) of 2AA at 1.73 µg/L, which was higher than in all the sequential fermentations with indigenous non-*Saccharomyces* yeast. Similarly, Álvarez-Fernández et al. [13] reported that two *S. cerevisiae* strains produced higher amounts of 2AA than non-*Saccharomyces* yeast.

N-acetyl-L-tryptophan (N-TRP) is one of the metabolites related to TRP metabolism which are not produced via kynurenic pathways (Figure 1). It has been used as a stabiliser for human serum albumin [33]. N-TRP, along with N-acetyl-L-tyrosine, was the most abundant compound in the wines. To our knowledge, there is no information available on the metabolization of this component by wine yeasts during fermentations. The initial concentration of N-TRP in the grape juice was 82.32 µg/L. A significant increase in N-TRP in the resulting wines was observed for both the monoculture and sequential fermentations. In the wines, the concentrations of N-TRP increased due to the activity of all the yeasts and were significantly affected by the *Ms* (1154.26 µg/L), *Mc* (781.99 µg/L), and *Pk* (725.86 µg/L) yeasts in the sequential fermentations with *Sc* compared to the *Sc* control (221.10 µg/L). The behaviour of these three indigenous yeasts in producing this compound differed significantly between the two treatments. *Ms-Sc* (1154.26 µg/L) and *Mc-Sc* (781.99 µg/L) exhibited higher concentrations of N-TRP than in their monoculture fermentations (183.76–263.08 µg/L), while *Pk* showed the opposite behaviour: *Pk* fermentation (1354.85 µg/L) and *Pk-Sc* fermentation (725.86 µg/L). Furthermore, L-tryptophan ethyl ester (TRP-EE) was not detected in the Maraština grape juice, although it is present in some other white musts [18]. When fermentation reaches a higher alcohol concentration, yeast produces ethyl esters from TRP in a one-step reaction [21]. All the utilised indigenous yeasts produced similar TRP-EE concentrations in the monoculture (6.20–16.70 µg/L) and sequential fermentations (7.36–14.94 µg/L), with no significant differences between the two practices. Fernandez-Cruz et al. [17] reported that non-*Saccharomyces* strains synthesised TRP-EE more quickly than strains from the *Saccharomyces* genera. Since N-acetyl-L-tryptophan ethyl ester (N-TRP-EE) was not detected in wine, the increase in TRP-EE concentration likely did not occur through the esterification of free amino acids but rather through the deacetylation of N-TRP-EE. Conversely, 5-hydroxy-L-tryptophan (5OH-TRP) was detected in grape juice at a 1.47 µg/L concentration. The yeasts hydrolysed this initial concentration by the end of fermentation. They did not synthesise 5OH-TRP from TRP, which aligns with the literature data [34], which pointed to bacteria as the main producer, except for two yeasts from the *Metschnikowia* genus. No significant differences were observed between the monoculture and sequential fermentation using the indigenous yeasts (0.67–0.71 µg/L). It can be transformed in melatonin, but that was not the case in this study due to the absence of melatonin in the final experimental wines. This indicates a direct impact of these two indigenous yeasts, as 5OH-TRP was absent in the *Sc* control. Yilmaz and Gökmen [20] reported that *S. cerevisiae* and non-*Saccharomyces* yeasts did not produce 5OH-TRP; instead, they consumed as a nitrogen source in synthetic must fermentations.

Indole-3-ethanol (tryptophol) (TOL) is a known derivative of tryptophan present in wines made from both red and white cultivars [35]. All the tested yeasts (indigenous and controls) were able to increase the concentration of this component, with *Mp* showing the highest concentration (190.13 µg/L). The biosynthesis of TOL is positively correlated with ethanol stress-tolerant yeasts, which have an enhanced expression of genes related to TRP metabolism [3]. TOL can have a positive impact, imparting a flowery character to wines. In [21], the authors show that TOL can react with sulphur dioxide, an additive in winemaking, to yield the tryptophan-2-sulfonate (TOL-SO_3_H). The concentration of 5-hydroxyindole-3-acetic acid (5OH-IAA), a serine metabolite, in the grapes was 2.39 µg/L, which decreased in all the experimental wines without significant differences among the different species and fermentation types. Furthermore, 5OH-IAA was not detected in sequential fermentations with *Mp* and *Lt* yeasts. Similarly, Tudela et al. [36] reported the absence of 5OH-IAA in nine sparkling wines, probably due to the low limit of detection and quantification. Mercolini et al. [37] reported a significantly higher concentration (105 µg/L) of these compounds in white wine. Indole-3-carboxylic acid (ICA) is a compound only detected in monoculture fermentations with *Ms* (0.19 µg/L), primarily due to yeast activity under tryptophan metabolism. Like other indole derivates, ICA can contribute floral or even herbaceous notes to wine, depending on its concentration and interaction with other compounds [38]. The remaining indigenous yeasts utilised the entire content from grape juice (0.32 µg/L), with *Hg* being the only yeast observed to produce indole-3-carboxylic acid ethyl ester (ICA-EE), which was absent in the grape juice. Indole-3-acetic acid (IAA) can significantly influence the sensory characteristics of wine and is most prevalent in carboxylic acids [39]. Also, IAA is included in the synthesis of 2-aminoacetophenone. The initial concentration (1.78 µg/L) aligns with previously observed levels in musts from Kerner and Malvasia grapes (<5.0 µg/L) [40,41]. Contreras et al. [42] noted the yeast’s reliance on TRP for IAA production. However, in our study, all the yeasts increased the IAA concentration, but there were no significant differences between them or between the fermentation types. A similar trend was observed for its derivative, indole-3-acetic acid ethyl ester (IAA-EE), which also exhibited no significant impact. Arapitsas et al. [21] only found detectable IAA in red and rose wines, but not in white wines, while sulfonated indole acetic acid (IAA-SO_3_H) was detected only in white wines; they concluded that this reaction was preferred in white wine. Conversely, our results showed higher concentrations of IAA than IAA-SO_3_H, indicating that sterile Maraština must is not a preferred medium for this conversion.

The metabolisms of phenylalanine (PHE) and tyrosine (TYR) are considered together. At the start of fermentation, phenylalanine (PHE) enhances floral and fruity wine aromas. Maraština grapes were characterised by a concentration of 2975.55 µg/L. Scutarașu et al. [43] reported an initial reduction in PHE concentration, followed by an increase after the middle of the alcoholic fermentation. PHE is known as one of the first amino acids yeast consumes during alcoholic fermentation [4]. Conversely, the result from this study indicated that the indigenous non-*Saccharomyces* strains better utilised TRP than PHE and TYR. The only significant impact of monoculture and sequential fermentation was observed for the *Hg* strain, which, in interaction with *Sc*, metabolised PHE concentrations (210.52 µg/L) that were more than three times lower. Furthermore, the indigenous *Lt* strain resulted in lower concentrations (113.07 µg/L) at the end of sequential fermentation compared to the commercial *Lt* Octave (272.88 µg/L). A similar trend was observed for *Mp* compared with the control *Mp* Flavia in both inoculation practices, where *Mp* in the monoculture fermentation consumed the best TYR (50.37 µg/L). N-acetyl-L-phenylalanine (N-PHE), an acetyl derivative of PHE, was present at 3.59 µg/L in grape juice. A significant impact of the sequential fermentations was observed for *Ms-Sc* (22.02 µg/L), resulting in a concentration that was twice as high compared to monoculture. N-acetyl derivatives of phenylalanine serve as precursors to important aroma compounds in wine, such as ethyl esters of PHE. TYR is produced from the amino acid PHE. The Maraština grapes contained 1932.57 µg/L of TYR. A significant effect of TYR in wine was evident in the fermentations with *Hu*, where it detected 222.52 µg/L compared to *Sc*. The *Hu* fermentation had a statistically higher remaining concentration on its own compared to its sequential fermentation with *Sc*. By the end of fermentation, the TYR concentration decreased approximately tenfold. One of its derivatives, tyrosol, is a higher alcohol. In our previous study [44], tyrosol was identified as the most abundant phenol in Maraština wines produced by spontaneous fermentation, although it did not show a significant correlation with the present microbiota, highlighting the Maraština variety as a source of tyrosol. However, in the current study, tyrosol was not quantified in either grape juice or any of the final wines, probably because the grapes were obtained from other locations that had different characteristics. The concentration of tyramine (TYRA), formed by the decarboxylation of TYR, decreased during fermentation from an initial concentration of 5.82 µg/L, probably in response to acid stress [20]. High levels of TYRA can have negative health effects; so, yeast typically works to reduce its content [45]. N-acetyl derivatives of tyrosine, specifically N-acetyl-L-tyrosine (N-TYR), increased during fermentation despite an initial concentration of 4.88 µg/L. This trend contrasts with the behaviour of ethyl ester analogues, probably due to the enzymatic deacetylation processes reported by Kradolfer et al. [46], which are involved in the production of TOL from TRP. *Pk* (753.04 µg/L) showed significant differences compared to *Sc* (97.57 µg/L). Interestingly, all the non-*Saccharomyces* isolates produced higher concentrations of N-TYR compared to *Sc*. As already pointed out in the beginning, the content of amino acids and their metabolites is largely determined by the grape variety [27,28]. Maraština is considered an autochthonous Croatian variety, but Šimon et al. [47] reported its high similarity to the globally known Malvasia del Chianti (Italy) and Pavlos (Greece). Therefore, these isolates can be expected to have similar behaviour in these two varieties.

### 3.2. Multivariant Analysis

**Figure 2:**
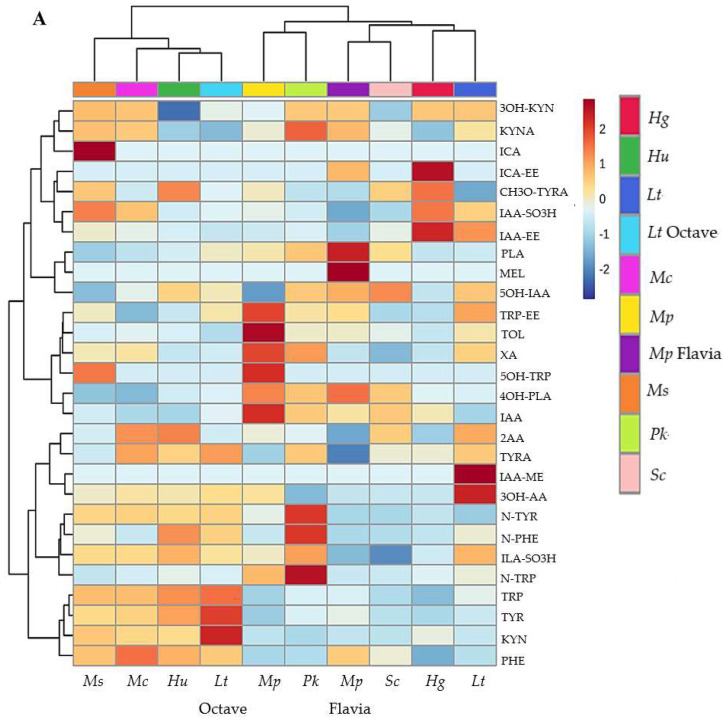

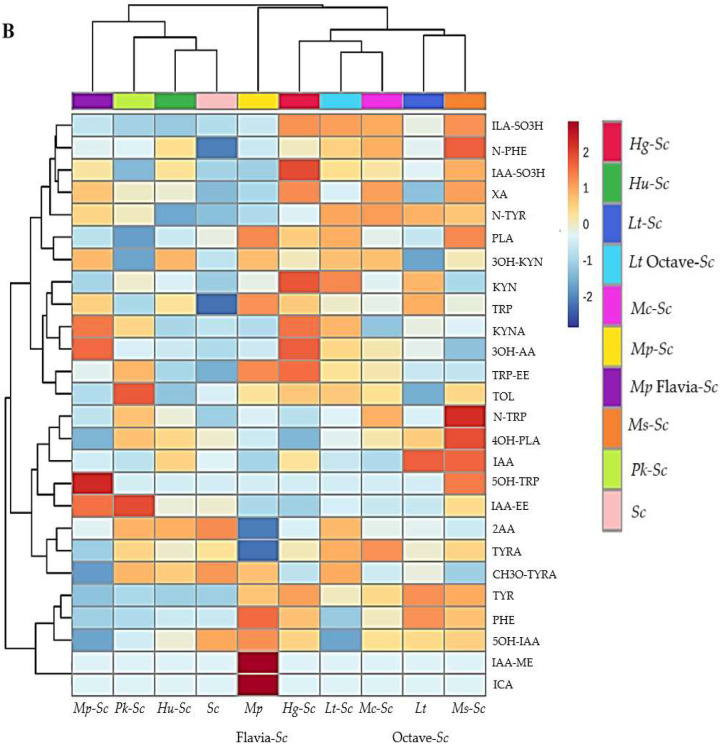
Hierarchical clustering representation corresponding to the 26 compounds related to TRP, PHE, and TYR metabolism in Maraština wines from monoculture (**A**) and sequential fermentations (**B**) obtained by UHPLC-QqQ-MS/MS analysis. The rows in the heatmap represent compounds, and the columns indicate yeast strains. Compounds are designated by abbreviations listed in Table 1. The relative content is illustrated through a chromatic scale from minimum (dark blue) to maximum (dark red).

Hierarchical clustering analysis performed on the two UHPLC-QqQ-MS/MS datasets obtained from the monoculture (Figure 2A) and sequential fermentations (Figure 2B) confirmed the discrimination of the final wines among the different inoculants. In monoculture fermentation, yeasts can be classified into three groups based on the production and/or utilisation of 26 metabolites, as follows: (i) *Ms*, *Mc*, *Hu*, and *Lt* Octave; (ii) *Mp* and *Pk*; and (iii) *Lt*, *Hg*, *Sc*, and *Mp* Flavia. It is evident that native yeasts, except for *Lt* and *Hg* in the third group, were not grouped with the control *Sc* yeast, while *Mp* and *Lt* were grouped differently from their commercial controls. The impact of sequential fermentation with *Sc* resulted in different groupings compared to the monoculture fermentations.

Furthermore, four groups were formed as follows: (i) *Mp-Sc*, *Pk-Sc*, *Hu-Sc*, and *Sc*; (ii) *Mp* Flavia-*Sc*; (iii) *Hg-Sc*, *Lt-Sc*, and *Mc-Sc*; and (iv) *Lt* Octave-*Sc* and *Ms-Sc*. We did not find a trend of influence based on the diversity of the origin of the isolates or the genus, considering that we had several species from certain genera (*Hanseniaspora* and *Metschnikowia*). It is concluded that each strain’s effects vary depending on whether it is inoculated alone or co-inoculated with *S. cerevisiae*.

**Figure 3:**
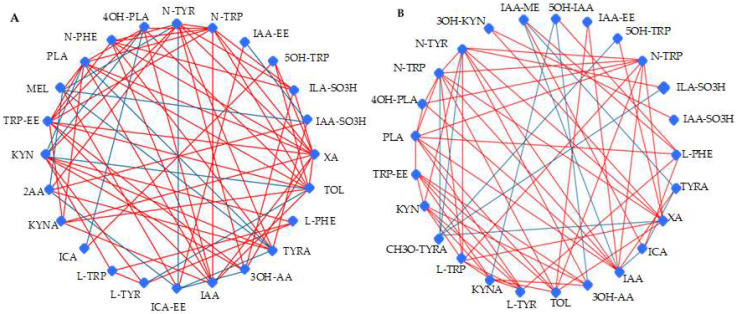
Debiased sparse partial correlation (DSPC) network illustrating the correlation between metabolites in monoculture fermentations (**A**) and sequential fermentations (**B**). The red lines indicate a positive correlation, while the blue line represents a negative correlation. Abbreviations are listed in Table 1.

It was also unnecessary to analyse the pathway, as the study applied a targeted approach to determine the compounds related to TRP, PHE, and TYR metabolism. Instead, we performed a debiased sparse partial correlation (DSPC) network analysis to determine the relationship between the metabolites analysed in the wines obtained from two fermentation practices (Figure 3). There was one subnetwork for monoculture (Figure 3A) with 24 different nodes and one for sequential fermentations (Figure 3B) with 25 nodes. The subnetworks provided a comprehensive picture, highlighting positive (red lines) and negative (blue lines) correlations among the metabolites.

## 4. Conclusions

This study focused on the extracellular metabolism of tryptophan, phenylalanine, and tyrosine to elucidate the role of indigenous non-*Saccharomyces* yeast metabolism using sterile Maraština grape juice in monoculture and sequential fermentation with *S. cerevisiae*. The results highlighted the behaviour of indigenous non-*Saccharomyces* yeasts due to their contribution to wine complexity throughout its aromatic amino acids metabolism. To the best of our knowledge, these are the first results related to TRP, PHE, and TYR metabolism for *M. sinensis/shanxiensis*, *M. chrysoperlae*, *H. guilliermondii*, and *L. thermotolerans* and for the indigenous yeasts. These three amino acids were the most abundant compounds in grape juice, but as expected, their concentrations decreased during alcoholic fermentation, resulting in the changed levels of the existing ones in grape juice and the production of other derivates, such as OH-ANT, TRP-EE, N-PHE, and ICE-EE. TRP was preferable over PHE and TYR for all indigenous non-*Saccharomyces* yeasts. Furthermore, the selection of inoculation practices significantly affected the concentration of tryptophan, N-acetyl tryptophan, tryptophol, kynurenic acid, 3-hydroxyanthranilic acid, xanthurenic acid, phenylalanine, and N-acetyl phenylalanine for certain yeast isolates. Based on inoculation practices and the UHPLC-MS/MS dataset at the end of the fermentations, the yeast isolates were grouped into different categories. It is concluded that each strain’s effects differ depending on whether it is inoculated individually or co-inoculated with *S. cerevisiae*. In particular, *H. guilliermondii*, *M. sinensis/shanxiensis*, and *M. pulcherrima*, with their behaviour during fermentation, were highlighted as yeasts with a major impact on the metabolites related to wine aroma and antioxidant properties, mainly through the TRP pathway. Further research on non-*Saccharomyces* isolates in alcoholic fermentation under real conditions is necessary to confirm their potential as starter cultures for improving wine characteristics.

## Figures and Tables

**Table 1:** Concentrations (µg/L) of aromatic amino acids and their derivatives quantified in Maraština grape juice and wines produced with non-*Saccharomyces* isolates in monoculture and sequential fermentations with commercial *Saccharomyces cerevisiae* EC 1118.

Compound (Abbreviation)	Grape Juice	T	Yeast									
			*M. chrysoperlae* K-11	*M.sinensis/* *shanxiensis* P-7	*M. pulcherrima* K-6	*L. thermotolerans* P-25	*H. uvarum* Z-7	*H.* *guilliermondii* N-29	*P. kluyveri* Z-3	*S. cerevisiae* EC 1118	*L. thermotolerans* Octave	*M. pulcherrima* Flavia
Tyrosol (TYR-OH)	nd	m	nd	nd	nd	nd	nd	nd	nd	nd	nd	nd
		s	nd	nd	nd	nd	nd	nd	nd		nd	nd
Tryptophol sulfonated (TOL-SO_3_H)	nd	m	nd	nd	nd	nd	nd	nd	nd	nd	nd	nd
		s	nd	nd	nd	nd	nd	nd	nd		nd	nd
3-hydroxykynurenine (3OH-KYN)	6.90 ± 0.32	m	6.38 ± 0.18 ab	6.57 ± 0.07 b	6.20 ± 0.05 ab	6.35 ± 0.32 ab	nd	6.31 ± 0.12 ab	6.30 ± 0.25 ab	6.25 ± 0.09 ab	6.38 ± 0.12 ab	6.24 ± 0.03 ab
		s	6.16 ± 0 a	6.48 ± 0.02 ab	6.31 ± 0.15 ab	6.17 ± 0.01 a	6.49 ± 0.04 ab	6.40 ± 0.01 ab	nd		nd	6.26 ± 0.1 ab
L-Tyrosine (TYR)	1932.87 ± 810.14	m	178.46 ± 26.77 bcdef	170.24 ± 20.14 abcde	50.37 ± 1.34 a	94.39 ± 22.56 abcd	222.52 ± 14.16 ef	64.1 ± 23.19 ab	108.65 ± 18.82 abcde	78.20 ± 35.43 abc	300.94 ± 9.49 f	194.94 ± 42.76 cdef
		s	131.66 ± 23.52 abcde	151.52 ± 48.69 abcde	161.60 ± 118.28 abcd	117.92 ± 43.10 abcde	78.21 ± 21.13 abc	155.35 ± 27.08 abcde	83.26 ± 15.05 abcd		208.7 ± 78.54 def	201.1 ± 6.46 cdef
Tyramine (TYRA)	5.82 ± 3.13	m	1.95 ± 1.01 b	1.47 ± 0.51 ab	0.65 ± 0.21 ab	1.66 ± 0.49 b	1.59 ± 0.06 b	1.26 ± 0.51 ab	1.67 ± 0.32 b	1.80 ± 0.21 b	1.98 ± 0.26 b	nd
		s	1.67 ± 1.06 b	1.97 ± 0.19 b	0.97 ± 0.75 ab	1.56 ± 0.29 ab	1.12 ± 0.20 ab	1.68 ± 0.09 b	1.34 ± 0.31 ab		1.62 ± 0.39 b	0.13 ± 0.09 a
3-metoxy-*p*-tyramine (CH3O-TYRA)	34.41 ± 9.71	m	16.72 ± 3.85 a	17.22 ± 8.72 a	13.83 ± 6.93 a	6.50 ± 2.18 a	21.05 ± 5.61 a	22.17 ± 11.75 a	9.60 ± 5.16 a	16.5 ± 8.83 a	11.73 ± 2.44 a	10.4 ± 4.68 a
		s	8.41 ± 6.37 a	5.68 ± 3.57 a	2.36 ± 0.83 a	15.54 ± 3.45 a	13.70 ± 16.61 a	8.26 ± 7.37 a	15.01 ± 3.97 a		10.17 ± 8.04 a	17.51 ± 7.11 a
5-hydroxy-L-tryptophan (5OH-TRP)	1.47 ± 0.03	m	nd	0.71 ± 0.00 a	0.68 ± 0.01 a	nd	nd	nd	nd	nd	nd	nd
		s	nd	0.67 ± 0.03 a	0.67 ± 0.02 a	nd	nd	nd	nd		nd	nd
L-phenylalanine (PHE)	2997.55 ± 1037.42	m	203.04 ± 47.49 bcde	158.30 ± 42.98 abcde	74.99 ± 16.71 a	108.78 ± 23.38 abc	169.96 ± 29.60 abcd	68.33 ± 10.59 a	119.24 ± 20.14 abc	118.93 ± 50.64 abc	247.78 ± 54.98 de	152.69 ± 48.59 abcd
		s	160.94 ± 23.18 abcde	209.37 ± 69.79 cde	121.09 ± 24.13 abc	113.07 ± 38.82 abc	119.82 ± 29.61 abc	210.52 ± 11.05 cde	93.99 ± 12.80 ab		246.52 ± 42.09 de	272.88 ± 23.1 e
L-Kynurenine (KYN)	3.30 ± 0.68	m	4.69 ± 2.27 ab	5.34 ± 0.68 b	1.26 ± 0.18 a	2.17 ± 0.13 ab	4.49 ± 1.30 ab	2.88 ± 1.11 ab	1.73 ± 0.24 ab	1.21 ± 0.06 a	10.62 ± 5.00 c	1.40 ± 0.06 ab
		s	1.51 ± 0.44 ab	1.27 ± 0.09 ab	1.97 ± 0.39 ab	2.08 ± 0.50 ab	1.48 ± 0.46 ab	2.27 ± 0.46 ab	1.59 ± 0.05 ab		1.91 ± 0.11 ab	1.55 ± 0.19 ab
3-hydroxyanthranilic acid (OH-ANT)	nd	m	65.66 ± 7.94 abc	58.24 ± 1.70 abc	64.56 ± 2.62 abc	69.5 ± 31.03 abc	43.09 ± 14.98 abc	34.52 ± 5.91 ab	38.54 ± 4.36 abc	34.55 ± 3.33 ab	62.62 ± 5.84 abc	33.92 ± 4.05 ab
		s	56.24 ± 52.21 abc	27.27 ± 0.62 a	87.01 ± 21.91 bc	81.99 ± 16.99 bc	42.01 ± 28.17 abc	89.76 ± 6.51 c	44.91 ± 5.72 abc		48.53 ± 0.81 abc	42.28 ± 12.62 abc
5-hydroxyindole-3-acetic acid (5OH-IAA)	2.39 ± 1.33	m	1.14 ± 0.39 a	1.13 ± 0.23 a	1.23 ± 0.32 a	1.36 ± 0.57 a	1.32 ± 0.14 a	1.03 ± 0.38 a	1.36 ± 0.46 a	1.52 ± 0.35 a	1.22 ± 0.35 a	1.43 ± 0.27 a
		s	1.12 ± 0.55 a	1.25 ± 0.21 a	nd	nd	1.11 ± 0.57 a	1.71 ± 0.13 a	1.07 ± 0.23 a		1.18 ± 0.01 a	1.66 ± 0.59 a
L-tryptophan (TRP)	2124.93 ± 616.84	m	0.06 ± 0.01 a	0.06 ± 0.01 a	0.02 ± 0.00 a	0.04 ± 0.01 a	0.07 ± 0.02 a	0.02 ± 0.01 a	0.04 ± 0.01 a	0.03 ± 0.01 a	0.08 ± 0.01 a	0.07 ± 0.04 a
		s	48.37 ± 11.39 ab	49.14 ± 18.58 ab	64.89 ± 42.28 b	52.02 ± 16.30 b	58.54 ± 31.45 b	66.64 ± 9.28 b	41.72 ± 9.46 ab		75.03 ± 37.83 b	81.38 ± 3.35 b
L-Tryptophan-d5 (TRP-d5)	1183.84 ± 267.28	m	1539.45 ± 26.37 a	1551.87 ± 56.06 a	1560.72 ± 31.34 a	1582.93 ± 44.96 a	1518.71 ± 1.86 a	1516.34 ± 13.27 a	1597.63 ± 42.92 a	1298.32 ± 355.5 a	1494.93 ± 59.29 a	1269.55 ± 582.76 a
		s	1515.22 ± 75.64 a	1460.44 ± 12.47 a	1548.41 ± 39.88 a	1559.78 ± 45.44 a	1477.84 ± 115.91 a	1616.09 ± 101.90 a	1537.51 ± 18.79 a		1430.72 ± 63.17 a	1344.15 ± 322.81 a
Xanturenic acid (XA)	13.41 ± 5.89	m	9.89 ± 1.44 abc	9.43 ± 1.66 abc	17.12 ± 2.00 d	11.21 ± 3.44 abcd	6.57 ± 1.25 ab	6.51 ± 1.90 ab	13.72 ± 4.95 cd	5.23 ± 2.12 a	7.13 ± 0.57 abc	6.41 ± 3.16 ab
		s	12.9 ± 1.53 bcd	17.48 ± 2.22 d	11.23 ± 2.19 abcd	11.27 ± 2.28 bcd	12.99 ± 2.10 bcd	13.68 ± 2.42 cd	8.74 ± 0.35 abc		5.53 ± 0.29 a	7.40 ± 0.35 abc
5-hydroxytryptohol (5OH-IET)	nd	m	nd	nd	nd	nd	nd	nd	nd	nd	nd	nd
		s	nd	nd	nd	nd	nd	nd	nd		nd	nd
Kyunurenic acid (KYNA)	3.29 ± 2.54	m	135.15 ± 13.11 abc	139.36 ± 17.84 abc	113.05 ± 0.58 abc	122.82 ± 57.13 abc	78.42 ± 58.25 ab	73.95 ± 22.11 a	171.77 ± 23.31 abcd	107.85 ± 58.80 ab	69.53 ± 24.87 a	170.28 ± 71.33 abcd
		s	96.79 ± 17.91 ab	139.73 ± 30.89 abc	299.93 ± 83.61 d	244.63 ± 66.65 cd	84.96 ± 21.59 ab	303.24 ± 13.00 d	211.82 ± 47.43 bcd		155.12 ± 10.63 abc	141 ± 37.63 abc
Indole-3-lactic acid-sulfonated (ILA-SO3H)	5.09 ± 4.96	m	3.02 ± 0.23 a	3.53 ± 1.72 a	2.80 ± 0.15 a	3.27 ± 0.66 a	3.29 ± 1.15 a	2.52 ± 0.95 a	3.38 ± 0.68 a	1.77 ± 0.12 a	2.94 ± 0.83 a	2.07 ± 0.54 a
		s	3.17 ± 0.63 a	3.36 ± 1.03 a	2.23 ± 1.31 a	3.26 ± 1.79 a	2.08 ± 1.32 a	4.68 ± 2.41 a	1.83 ± 1.17 a		2.3 ± 0.30 a	1.95 ± 0.43 a
Indole-3-acetic acid-sulfonated (IAA-SO3H)	2.37 ± 1.71	m	1.59 ± 0.61 a	1.98 ± 0.32 a	1.33 ± 0.53 a	1.51 ± 0.91 a	1.08 ± 0.68 a	2.03 ± 0.70 a	0.95 ± 1.19 a	1.03 ± 0.07 a	1.04 ± 0.99 a	0.34 ± 0.33 a
		s	1.17 ± 0.28 a	1.46 ± 0.44 a	1.18 ± 0.21 a	1.34 ± 0.81 a	1.20 ± 0.39 a	1.84 ± 0.62 a	0.56 ± 0.12 a		0.98 ± 0.73 a	1.03 ± 0.19 a
L-Tryptophan ethyl ester (TRP-EE)	nd	m	10.46 ± 1.26 a	9.32 ± 1.42 a	16.70 ± 1.58 a	13.07 ± 3.76 a	7.08 ± 1.67 a	6.20 ± 1.55 a	9.98 ± 2.35 a	10.29 ± 5.70 a	9.81 ± 0.68 a	10.48 ± 5.70 a
		s	10.61 ± 7.23 a	8.25 ± 1.16 a	13.74 ± 4.62 a	14.61 ± 5.12 a	7.36 ± 4.56 a	14.94 ± 1.75 a	12.76 ± 0.71 a		11.34 ± 1.45 a	14.08 ± 0.14 a
Indole 3-lactic acid (ILA)	nd	m	nd	nd	nd	nd	nd	nd	nd	nd	nd	nd
		s	nd	nd	nd	nd	nd	nd	nd		nd	nd
3-(4-hydroxyphenyl) lactic acid (4 OH-PLA)	3.73 ± 1.18	m	32.08 ± 2.64 a	25.95 ± 4.10 a	99.44 ± 6.04 a	50.74 ± 6.23 a	55.28 ± 27.67 a	52.44 ± 31.92 a	79.45 ± 25.71 a	90.16 ± 48.26 a	51.24 ± 0.78 a	104.97 ± 43.65 a
		s	80.41 ± 10.92 a	103.83 ± 6.28 a	58.55 ± 22.96 a	75.03 ± 31.54 a	84.16 ± 29.81 a	59.12 ± 11.16 a	87.96 ± 9.38 a		86.14 ± 11.39 a	70.88 ± 48.96 a
N-acetyl-L-tyrosine (N-TYR)	4.88 ± 0.92	m	410.54 ± 42.29 abcd	397.13 ± 130.23 abcd	257.89 ± 84.41 abc	594.20 ± 114.90 cd	387.87 ± 104.42 abcd	159.46 ± 2.28 ab	753.04 ± 92.54 d	97.57 ± 3.34 a	404.52 ± 15.36 abcd	97.22 ± 13.65 a
		s	493.64 ± 123.32 bcd	417.82 ± 30.28 abcd	493.29 ± 190.97 bcd	573.47 ± 276.20 cd	425.06 ± 239.88 abcd	428.76 ± 109.83 abcd	315.83 ± 4.61 abc		454.39 ± 31.13 abcd	301.18 ± 196.65 abc
Indole-3-carboxylic acid (ICA)	0.32 ± 0.19	m	nd	0.19 ± 0.14	nd	nd	nd	nd	nd	nd	nd	nd
		s	nd	nd	nd	nd	nd	nd	nd		nd	0.02 ± 0.02
Cinnamoyl glycine (CYG)	nd	m	nd	nd	nd	nd	nd	nd	nd	nd	nd	nd
		s	nd	nd	nd	nd	nd	nd	nd		nd	nd
N-acetyl-L-tyrosine ethyl ester (N-TYR-EE)	nd	m	nd	nd	nd	nd	nd	nd	nd	nd	nd	nd
		s	nd	nd	nd	nd	nd	nd	nd		nd	nd
5-methoxytryptophol (5ME-IET)	nd	m	nd	nd	nd	nd	nd	nd	nd	nd	nd	nd
		s	nd	nd	nd	nd	nd	nd	nd		nd	nd
Melatonine (MEL)	nd	m	nd	nd	nd	nd	nd	nd	nd	nd	nd	0.31 ± 0.23
		s	nd	nd	nd	nd	nd	nd	nd		nd	nd
Indole-3-ethanol (tryptophol) (TOL)	0.41 ± 0.10	m	42.64 ± 2.43 b	37.20 ± 7.72 ab	190.13 ± 22.53 c	63.42 ± 14.08 ab	36.44 ± 16.4 ab	32.07 ± 9.29 a	77.95 ± 7.17 ab	45.42 ± 21.40 ab	21.79 ± 1.71 a	54.6 ± 25.12 ab
		s	55.94 ± 23.25 ab	58.09 ± 7.36 ab	57.75 ± 20.05 ab	60.73 ± 22.48 ab	47.83 ± 5.83 ab	60.88 ± 9.35 ab	78.17 ± 3.03 b		42.34 ± 4.79 ab	55.14 ± 2.65 ab
Indole-3-acetic acid (IAA)	1.78 ± 0.82	m	6.49 ± 0.52 ab	5.49 ± 1.18 ab	10.42 ± 1.73 ab	7.23 ± 1.85 ab	4.61 ± 0.83 a	6.54 ± 3.91 ab	10.34 ± 2.53 ab	7.48 ± 2.84 ab	8.41 ± 0.08 ab	6.74 ± 3.10 ab
		s	9.23 ± 1.45 ab	11.66 ± 1.87 b	7.08 ± 2.81 ab	6.88 ± 2.13 ab	9.16 ± 2.13 ab	8.69 ± 1.11 ab	9.53 ± 0.98 ab		11.77 ± 1.17 b	6.50 ± 4.25 ab
Indole-3-propionic acid (IPA)	nd	m	nd	nd	nd	nd	nd	nd	nd	nd	nd	nd
		s	nd	nd	nd	nd	nd	nd	nd		nd	nd
2-aminoacetophenone (2AA)	0.96 ± 0.7	m	2.26 ± 0.81 a	0.95 ± 0.44 a	1.85 ± 0.48 a	2.07 ± 0.65 a	2.37 ± 0.46 a	0.82 ± 0.59 a	1.06 ± 0.23 a	1.73 ± 0.43 a	nd	nd
		s	1.27 ± 1 a	1.17 ± 0.71 a	1.67 ± 0.28 a	1.59 ± 0.77 a	1.63 ± 0.19 a	1.21 ± 0.69 a	1.62 ± 2.01 a		1.80 ± 0.59 a	0.67 ± 0.25 a
N-acetyl-L-tryptophan ethyl ester (N-TRP-EE)	nd	m	nd	nd	nd	nd	nd	nd	nd	nd	nd	nd
		s	nd	nd	nd	nd	nd	nd	nd		nd	nd
Indole-3-acetic acid methyl ester (IAA-ME)	nd	m	nd	nd	nd	nd	nd	nd	nd	nd	nd	nd
		s	nd	nd	nd	nd	nd	nd	nd		nd	0.1 ± 0.02
Indole-3-butyric acid (IBA)	nd	m	nd	nd	nd	nd	nd	nd	nd	nd	nd	nd
		s	nd	nd	nd	nd	nd	nd	nd		nd	nd
N-acetyl-L-phenylalanine (N-PHE)	nd	m	11.7 ± 1 abc	10.20 ± 1.93 ab	13.04 ± 1.11 abc	14.61 ± 0.13 abc	14.81 ± 1.64 abc	11.17 ± 1.26 abc	17.92 ± 2.93 abc	7.57 ± 3.43 ab	12.26 ± 0.13 abc	7.27 ± 3.65 a
		s	19.04 ± 3.1 bc	22.02 ± 1.35 c	14.42 ± 6.78 abc	17.49 ± 6.39 abc	16.76 ± 5.30 abc	15.67 ± 0.74 abc	14.31 ± 1.09 abc		14.54 ± 1.14 a	13.33 ± 9.72 abc
Phenyllactic acid (PLA)	12.74 ± 5.02	m	83.85 ± 7.14 a	64.15 ± 22.56 a	190.81 ± 19.41 ab	147.28 ± 38.93 ab	97.94 ± 31.86 a	87.92 ± 43.36 a	155.86 ± 37.88 ab	139.52 ± 78.13 ab	121.21 ± 5.32 ab	248.40 ± 113.08 b
		s	137.67 ± 25.15 ab	174.47 ± 14.94 ab	124.98 ± 37.06 ab	165.93 ± 86.67 ab	129.94 ± 10.60 ab	156.98 ± 47.7 ab	155.53 ± 19.21 ab		175.94 ± 21.06 ab	174.58 ± 12.20 ab
N-acetyl-L-tryptophan (N-TRP)	82.23 ± 58.48	m	263.08 ± 62.27 ab	183.76 ± 41.87 a	711.85 ± 59.99 bcd	448.51 ± 248.87 abc	339.09 ± 83.00 abc	394.55 ± 286.28 abc	1354.85 ± 220.49 e	221.10 ± 96.34 a	273.74 ± 21.52 ab	252.31 ± 105.44 ab
		s	781.99 ± 275.57 cd	1154.26 ± 94.28 de	332.36 ± 171.93 abc	440.95 ± 193.75 abc	497.32 ± 197.54 abc	309.73 ± 44.62 abc	725.86 ± 90.17 bcd		411.88 ± 18.93 abc	424.69 ± 248.3 abc
Indole-3-carboxylic acid ethyl ester (ICA-EE)	nd	m	nd	nd	nd	nd	nd	1.76 ± 0.48	nd	nd	nd	1.38 ± 0.62
		s	nd	nd	nd	nd	nd	nd	nd		nd	nd
Indole-3-acetic acid ethyl ester (IAA-EE)	0.76 ± 0.14	m	0.95 ± 0.32 a	1.11 ± 0.48 a	0.92 ± 0.10 a	2.12 ± 2.04 a	1.10 ± 0.32 a	3.06 ± 2.08 a	1.18 ± 0.09 a	0.97 ± 0.33 a	0.99 ± 0.27 a	2.9 ± 2.26 a
		s	1.55 ± 0.91 a	1.13 ± 0.16 a	1.59 ± 1.27 a	0.83 ± 0.13 a	0.95 ± 0.08 a	0.91 ± 0.09 a	1.73 ± 1.37 a		1.10 ± 0.04 a	0.82 ± 0.19 a

Data are representative mean ± standard deviation of three biological replications. Different letters in the rows represent statistically significant differences between yeasts at the significance level of *p* < 0.05, separately for two fermentation practices (two-way ANOVA and Tukey test). Different letters in the columns represent statistically significant differences between fermentation treatments at the significance level of *p* < 0.05. Abbreviations: nd—not detected; T—inoculation practice; m—monoculture fermentation; s—sequential fermentations.

## Data Availability

The original contributions presented in the study are included in the article/Supplementary Material, further inquiries can be directed to the corresponding author.

## Supplementary Materials

The following supporting information can be downloaded at: https://www.mdpi.com/article/10.3390/foods13182939/s1, Table S1: Multiple reaction monitoring parameters and retention time for metabolites related to amino acid metabolism.

## References

[1] Fleet G. (2003). Yeast interactions and wine flavour. Int. J. Food Microbiol..

[2] Bell S.-J., Henschke P.A. (2005). Implications of nitrogen nutrition for grapes, fermentation and wine. Aust. J. Grape Wine Res..

[3] Engin A., Engin A., Engin A.B. (2015). Wine Flavor and Tryptophan. Tryptophan Metabolism: Implications for Biological Processes, Health and Disease; Molecular and Integrative Toxicology.

[4] Crépin L., Nidelet T., Sanchez I., Dequin S., Camarasa C. (2012). Sequential use of nitrogen compounds by *Saccharomyces cerevisiae* during wine fermentation: A model based on kinetic and regulation characteristics of nitrogen permeases. Appl. Environ. Microbiol..

[5] Sprenger J., Hardeland R., Fuhrberg B., Han S.Z. (1999). Melatonin and other 5-methoxylated indoles in yeast: Presence in high concentrations and dependence on tryptophan availability. Cytologia.

[6] Rodríguez-Naranjo M.I., Torija M.J., Mas A., Cantos-Villar E., García-Parrilla M.C. (2012). Production of melatonin by *Saccharomyces* strains under growth and fermentation conditions. J. Pineal Res..

[7] Liang Z., Fang Z., Pai A., Luo J., Gan R., Gao Y., Zhang P. (2022). Glycosidically bound aroma precursors in fruits: A comprehensive review. Crit. Rev. Food Sci. Nutr..

[8] Milanović V., Cardinali F., Boban A., Gajdoš Kljusurić J., Osimani A., Aquilanti L., Garofalo C., Budić-Leto I. (2023). White grape variety Maraština as a promising source of non-*Saccharomyces* yeasts intended as starter cultures. Food Biosci..

[9] Jolly N.P., Varela C., Pretorius I.S. (2014). Not your ordinary yeast: Non-*Saccharomyces* yeasts in wine production uncovered. FEMS Yeast Res..

[10] García V., Vásquez H., Fonseca F., Manzanares P., Viana F., Martínez C., Ganga M.A. (2010). Effects of using mixed wine yeast cultures in the production of Chardonnay wines. Rev. Argent. Microbiol..

[11] Belda I., Ruiz J., Esteban-Fernández A., Navascués E., Marquina D., Santos A., Moreno-Arribas M.-V. (2017). Microbial contribution to Wine aroma and its intended use for Wine quality improvement. Molecules.

[12] Vejarano R., Gil-Calderón A. (2021). Commercially Available Non-*Saccharomyces* Yeasts for Winemaking: Current Market, Advantages over *Saccharomyces*, Biocompatibility, and Safety. Fermentation.

[13] Álvarez-Fernández M.A., Fernandez-Cruz E., García Parrilla M.C., Troncoso A.M., Mattivi F., Vrhovsek U., Arapitsas P. (2019). *Saccharomyces cerevisiae* and *Torulaspora delbrueckii* intra- and extra-cellular aromatic amino acids metabolism. J. Agric. Food Chem..

[14] Swiegers J.H., Bartowsky E.J., Henschke P.A., Pretorius I.S. (2005). Yeast and bacterial modulation of wine aroma and flavour. Aust. J. Grape Wine Res..

[15] Álvarez-Fernández M.A., Carafa I., Vrhovsek U., Arapitsas P. (2020). Modulating Wine Aromatic Amino Acid Catabolites by Using *Torulaspora delbrueckii* in Sequentially Inoculated Fermentations or *Saccharomyces Cerevisiae* Alone. Microorganisms.

[16] Hoenicke K., Simat T.J., Steinhart H., Christoph N., Geßner M., Köhler H.-J. (2002). “Untypical aging off-flavor” in wine: Formation of 2-aminoacetophenone and evaluation of its influencing factors. Anal. Chim. Acta.

[17] Fernández-Cruz E., Alvarez-Fernández M.A., Valero E., Troncoso A.M., García-Parrilla M.C. (2016). Melatonin and derived tryptophan metabolites produced during alcoholic fermentation by different yeast strains. Food Chem..

[18] Fernández-Cruz E., Cerezo A.B., Cantos-Villar E., Troncoso A.M., García-Parrilla M.C. (2018). Time course of l-tryptophan metabolites when fermenting natural grape musts: Effect of inoculation treatments and cultivar on the occurrence of melatonin and related indolic compounds. Aust. J. Grape Wine Res..

[19] González B., Vázquez J., Morcillo-Parra M.A., Mas A., Torija M.-J., Beltran G. (2018). The production of aromatic alcohols in non-*Saccharomyces* wine yeast is modulated by nutrient availability. Food Microbiol..

[20] Yılmaz C., Gökmen V. (2021). Formation of amino acid derivatives in white and red wines during fermentation: Effects of non-*Saccharomyces* yeasts and *Oenococcus oeni*. Food Chem..

[21] Arapitsas P., Guella G., Mattivi F. (2018). The impact of SO_2_ on wine flavanols and indoles in relation to wine style and age. Sci. Rep..

[22] Anesi A., Berding K., Clarke G., Stanton C., Cryan J.F., Caplice N., Ross R.P., Doolan A., Vrhovsek U., Mattivi F. (2022). Metabolomic Workflow for the Accurate and High-Throughput Exploration of the Pathways of Tryptophan, Tyrosine, Phenylalanine, and Branched-Chain Amino Acids in Human Biofluids. J. Proteome Res..

[23] MetaboAnalyst 5.0. https://www.metaboanalyst.ca/MetaboAnalyst/ModuleView.xhtml.

[24] Basu S., Duren W., Evans C.R., Burant C.F., Michailidis G., Karnovsky A. (2017). Sparse network modeling and metscape-based visualization methods for the analysis of large-scale metabolomics data. Bioinformatics.

[25] Dei Cas M., Vigentini I., Vitalini S., Laganaro A., Iriti M., Paroni R., Foschino R. (2021). Tryptophan Derivatives by *Saccharomyces cerevisiae* EC1118: Evaluation, Optimization, and Production in a Soybean-Based Medium. Int. J. Mol. Sci..

[26] Erhardt S., Olsson S.K., Engberg G. (2009). Pharmacological Manipulation of Kynurenic Acid. CNS Drugs.

[27] Rongvaux A., Andris F., Van Gool F., Leo O. (2003). Reconstructing eukaryotic NAD metabolism. BioEssays.

[28] Gutiérrez-Gamboa G., Carrasco-Quiroz M., Martínez-Gil A.M., Pérez-Alvarez E.P., Garde-Cerdán T., Moreno-Simunovic Y. (2018). Grape and wine amino acid composition from Carignan noir grapevines growing under rainfed conditions in the Maule Valley, Chile: Effects of location and rootstock. Food Res. Int..

[29] Gutiérrez-Gamboa G., Portu J., López R., Santamaría P., Garde-Cerdán T. (2018). Effects of a combination of elicitation and precursor feeding on grape amino acid composition through foliar applications to Garnacha vineyard. Food Chem..

[30] Lovelace M.D., Varney B., Sundaram G., Lennon M.J., Lim C.K., Jacobs K., Brew B.J. (2017). Recent evidence for an expanded role of the kynurenine pathway of tryptophan metabolism in neurological diseases. Neuropharmacology.

[31] Turska M., Rutyna R., Paluszkiewicz M., Terlecka P., Dobrowolski A., Pelak J., Turski M.P., Muszyńska B., Dabrowski W., Kocki T. (2018). Presence of kynurenic acid in alcoholic beverages—Is this good news, or bad news?. Med. Hypotheses.

[32] Lugo-Huitrón R., Muñiz P.U., Pineda B., Pedraza-Chaverrí J., Ríos C., Pérez-de la Cruz V. (2013). Quinolinic acid: An endogenous neurotoxin with multiple targets. Oxid. Med. Cell Longev..

[33] Hogan K.L., Leiske D., Salisbury C.M. (2017). Characterization of N-Acetyl-Tryptophan Degradation in Protein Therapeutic Formulations. J. Pharm. Sci..

[34] Zhang Z., Yu Z., Wang J., Yu Y., Li L., Sun P., Fan X., Xu Q. (2022). Metabolic engineering of *Escherichia coli* for efficient production of L-5-hydroxytryptophan from glucose. Microb. Cell Fact..

[35] Gil C., Gómez-Cordovés C. (1986). Tryptophol content of young wines made from Tempranillo, Garnacha, Viura and Airén grapes. Food Chem..

[36] Tudela R., Ribas-Agustí A., Buxaderas S., Riu-Aumatell M., Castellari M., López-Tamames E. (2016). Ultrahigh-Performance Liquid Chromatography (UHPLC)–Tandem Mass Spectrometry (MS/MS) Quantification of Nine Target Indoles in Sparkling Wines. J. Agric. Food Chem..

[37] Mercolini L., Mandrioli R., Raggi M.A. (2012). Content of Melatonin and Other Antioxidants in Grape-Related Foodstuffs: Measurement Using a MEPS-HPLC-F 295 Method. J. Pineal Res..

[38] Moreno-Arribas M.V., Polo M.C., Moreno-Arribas M.V., Polo M.C. (2009). Amino Acids and Biogenic Amines. Wine Chemistry and Biochemistry.

[39] Mas A., Guillamon J.M., Torija M.J., Beltran G., Cerezo A.B., Troncoso A.M., Garcia-Parrilla M.C. (2014). Bioactive compounds derived from the yeast metabolism of aromatic amino acids during alcoholic fermentation. Biomed Res. Int..

[40] Simat T.J., Hoenicke K., Gessner M., Christoph N. (2004). Metabolism of tryptophan and indole-3-acetic acid formation during vinification and its influence on the formation of 2-aminoacetophenone. Mitt. Klosterneuburg.

[41] Maslov L., Jeromel A., Herjavec S., Korenika A.M.J., Mihaljević Ž.M., Plavša T. (2011). Indole-3-acetic acid and tryptophan in Istrian Malvasia grapes and wine. J. Agric. Food Environ..

[42] Contreras A., Hidalgo C., Henschke P.A., Chambers P.J., Curtin C., Varela C. (2014). Evaluation of non-*Saccharomyces* yeasts for the reduction of alcohol content in wine. Appl. Environ. Microbiol..

[43] Scutarașu E.C., Luchian C.E., Cioroiu I.B., Trincă L.C., Cotea V.V. (2022). Increasing Amino Acids Content of White Wines with Enzymes Treatments. Agronomy.

[44] Boban A., Milanović V., Veršić Bratinčević M., Botta C., Ferrocino I., Cardinali F., Ivić S., Rampanti G., Budić-Leto I. (2024). Spontaneous fermentation of Maraština wines: The correlation between autochthonous mycobiota and phenolic compounds. Food Res. Int..

[45] del Rio B., Fernandez M., Redruello B., Ladero V., Alvarez M.A. (2024). New insights into the toxicological effects of dietary biogenic amines. Food Chem..

[46] Kradolfer P., Niederberger P., Htitter R. (1982). Tryptophan Degradation in *Saccharomyces cerevisiae*: Characterization of Two Aromatic Aminotransferases. Arch. Microbiol..

[47] Šimon S., Maletić E., Karoglan-Kontić J., Crespan M., Schneider A., Pejic I. (2007). Cv. Maraština—A New Member of Malvasia Group. II Simposio Internazionale “Malvasie del Mediterraneo”. https://www.bib.irb.hr/350268.

