# Supplementary material for: Modulation of Aromatic Amino Acid Metabolism by Indigenous Non-Saccharomyces Yeasts in Croatian Maraština Wines
Source: Foods. 2024 Sep 17;13(18):2939. doi: 10.3390/foods13182939 (PMC11431312; doi:10.3390/foods13182939)
Supplement: Supplementary file 1 [file foods-13-02939-s001.zip › foods-3193779-supplementary.pdf]

Table S1. Multiple Reaction Monitoring parameters and retention time for metabolites related to amino acid metabolism.

	Compound	RT (min)	quantifier MRM (collision energy)	qualifier MRM (collision energy)
1	Tyrosol (TYR-OH)	0.0	139.1→93(14)	139.1→102(20)
2	Tryptophol sulfonated (TOL-SO_3H)	2.5	240→160(-38)	240→130(-38)
3	3-hydroxykynurenine (3OH-KYN)	0.9	225.3→208.2(13)	225.3→162.1(25)
4	L-Tyrosine (TYR)	1.0	182.2→91.0(35)	182.2→119(31)
5	Tyramine (TYRA)	1.4	138.2→121.0(14)	138.2→77(36)
6	3-methoxy-p-tyramine (CH_3O-TYRA)	1.5	168.3→151.1(14)	168.3→91(32)
7	5-hydroxy-L-tryptophan (5OH-TRP)	1.6	221.3→162.2(25)	221.3→134.2(34)
8	L-Phenylalanine (PHE)	1.7	166.2→103(36)	166.2→77(49)
9	L-Kynurenine (KYN)	1.7	209.3→146(25)	209.3→94(19)
10	3-hydroxyanthranilic acid (OH-ANT)	1.9	154.2→136.1(15)	154.2→80(34)
11	5-hydroxyindole-3-acetic acid (5OH-IAA)	2.2	192.2→146.2(19)	192.2→91(48)
12	L-Tryptophan (TRP)	2.2	205.2→188.1(13)	205.2→146(24)
13	L-Tryptophan-d5 (IS)	2.2	210.3→192.1(14)	210.3→150(25)
14	Xanthurenic acid (XA)	2.3	206.0→160.0(25)	206→132(40)
15	5-hydroxytryptohol (5OH-IET)	2.3	178.2→160.1(20)	178.2→115(36)
16	Kynurenic acid (KYNA)	2.3	190.2→144.1(25)	190.2→89(52)
17	Indole-3-acetic acid-sulfonated (IAC-SO_3H)	2.53	254.1→130.1(-24)	254.1→210.1(-28)
18	Indole-3-lactic acid-sulfonated (ILA- SO_3H)	2.43	284.1→222.1(-18)	284.1→142(-26)
19	L-Tryptophan ethyl ester (TRP-EE)	2.6	233.3→216.3(14)	233.3→174.2(22)
20	Indole-3-lactic acid (ILA)	2.7	206.3→118.1(29)	206.3→13011(40)
21	3-(4-hydroxyphenyl) lactic acid (4OH-PLA)	2.7	181.2→163.1(-16)	181.2→135(-21)
22	N-acetyl-L-tyrosine (N-TYR)	2.2	222.1→180.1(-18)	222.1→163(-28)
23	Indole-3-carboxylic acid (ICA)	2.8	162.2→116.2(28)	162.2→91(33)
24	Cinnamoyl glycine (CYG)	2.8	206.1→131.0(18)	206.1→103(38)
25	N-acetyl-L-tyrosine ethyl ester (N-TYR-EE)	2.8	252.2→136.2(27)	252.2→178.2(16)
26	5-methoxytryptophol (5ME-IET)	2.9	192.3→174.3(20)	192.3→130.1(49)
27	Melatonin (MEL)	2.9	233.1→174.1(21)	233.1→159(37)
28	Indole-3-ethanol (tryptophol) (TOL)	3.0	162.3→144.1(19)	162.3→117.1(30)
29	Indole-3-acetic acid (IAA)	3.0	176.2→130(40)	176.2→102.9(19)
30	Indole-3-propionic acid (IPA)	3.2	190.2→130.1(24)	190.2→103.1(50)
31	2-aminoacetophenone (2AA)	3.2	136.0→117(14)	136→42(46)
32	N-acetyl-L-tryptophan ethyl ester (N-TRP-EE)	3.3	275.3→201.1(18)	275.3→229(13)
33	Indole-3-acetic acid methyl ester (IAA-ME)	3.5	190.2→130.2(25)	190.2→103(48)
34	Indole-3-butyric acid (IBA)	3.5	204.3→186.1(19)	204.3→130(35)
35	N-acetyl-L-phenylalanine (N-PHE)	2.7	206.1→164(-17)	206.1→147(-22)
36	Phenyllactic acid (PLA)	3.7	164.9→147(-15)	164.9→119(-19)
37	N-acetyl-L-tryptophan (N-TRP)	2.8	245.1→203.1(-18)	245.1→73.9(-23)
38	Indole-3-carboxylic acid ethyl ester (ICA-EE)	3.8	190.2→118.1(24)	190.2→162.1(16)
39	Indole-3-acetic acid ethyl ester (IAA-EE)	3.9	204.3→130.2(25)	204.3→103(50)
